# Acute Confusional Migraines: A Case Report

**DOI:** 10.5811/cpcem.4918

**Published:** 2024-05-14

**Authors:** Devin M. Howell, Garrett Lamouree

**Affiliations:** * Northwell Health, Department of Emergency Medicine, Huntington, New York; † Binghamton University, Binghamton, New York

**Keywords:** *pediatric*, *migraine*, *confusion*, *stroke*, *case report*

## Abstract

**Introduction:**

Acute confusional migraine (ACM) is a rare variant of migraine that is benign and self-resolving but difficult to diagnose. Without known causative pathophysiology and a lack of recognition in the International Classification of Headache Disorders (ICHD-3), ACM offers a puzzling clinical presentation. There currently is no standardized treatment for ACM, but with a growing anecdotal dataset there is the opportunity to formally recognize and establish protocols to improve patient care and outcomes.

**Case Report:**

A 14-year-old female presented to the emergency department (ED) with acute onset of confusion, vision changes, right-sided weakness, and dysarthria one hour prior to arrival. A stroke workup at the initial ED offered no pertinent findings. The patient was transferred to a pediatric specialty ED where all symptoms, aside from numbness and a mild headache, resolved during transfer. After administration of a migraine cocktail at the pediatric specialty ED, all remaining symptoms completely resolved. The patient was discharged home from the ED the same evening with outpatient follow-up.

**Conclusion:**

This case presents the difficulty of diagnosing and treating ACM prior to its self-resolution. It highlights the need for formal recognition of the condition by the ICHD-3. In doing so, greater recognition will promote more research, awareness, and establishment of a standardized treatment for ACM.

Population Health Research Capsule What do we already know about this clinical entity?

*Acute confusional migraine (ACM) can be treated with traditional migraine medications but may present with worrying symptoms, including significant neurological deficits.*

What makes this presentation of disease reportable?

*Acute confusional migraine is an under-reported stroke mimic that may present to the emergency department and should, therefore, be considered.*

What is the major learning point?

*Clinicians should be aware of ACM, particularly in children, to have a broader differential for those presenting with stroke-like symptoms.*

How might this improve emergency medicine practice?

*Through increased awareness of ACM, emergency physicians may be able to avoid unnecessary radiation and lab testing.*

## INTRODUCTION

Acute confusional migraine (ACM) afflicts all ages, with a predominance in children, and involves a suite of symptoms, including confusion, aphasia, and hemiplegia.^1^ With no known causative pathophysiology and a lack of recognition in the International Classification of Headache Disorders (ICHD-3) as a migraine variant, knowledge of this disorder is limited among clinicians, making ACM an exclusionary diagnosis.^1^ The clinical presentation of ACM offers a broad range of differential diagnoses, making it difficult to diagnose patients in the absence of an individual or family history of migraines.^2^ Together, these aspects have likely led to underdiagnosis of ACM and a lack of standardized treatment.^1^ ^,^ ^2^

We present the case of a 14-year-old female who presented to the emergency department (ED) with an acute onset of confusion, vision changes, right-sided weakness, and dysarthria, which began one hour prior to arrival. Her symptoms were preceded by an incident of minor head trauma that occurred the day prior. Both the rarity of ACM and the discussion regarding ACM-associated head trauma make this case a unique account of the disorder.

## CASE REPORT

A 14-year-old female presented to the ED with acute altered mental status. Per the mother, an hour prior to ED arrival, the patient had an acute onset of confusion with vision changes, followed by dysarthria and right-sided upper extremity weakness and numbness. It was discovered that the patient had suffered a minor head trauma from a backward fall the day prior without loss of consciousness or vomiting. She was not evaluated for her head trauma prior to the current visit. The patient had a history of intermittent headaches (not formally diagnosed) and a family history of one episode of transient global amnesia in her mother. There was no other reported medical history or any prior psychiatric history. Upon presentation to the ED, the patient was not oriented to self and could not follow commands. Physical examination (PE) was completed with the patient responding “I don’t know” to most questions and commands aside from her name, a mild right pronator drift, and inability to follow additional commands, thereby limiting her examination. All other PE findings were unremarkable, including any signs of external trauma.

A National Institute of Health Stroke Scale (NIHSS) was calculated to be 6, and a code stroke was called. Deficits included level of consciousness, in that she only answered one question correctly (1) and performed neither task for level of consciousness commands correctly (2). Her motor function was hindered with right arm drift, with the limb holding 90 (or 45) degrees, but drifting down before the full 10 seconds, but not hitting the bed or support (1). Her final deficit was severe aphasia (2), with all communication through fragmented expression, which required a great need for inference, questioning, and guessing by the listener. The range of information that could be exchanged was limited; listener carried the burden of communication. Examiner could not identify materials provided from patient response.

**Image 1.**
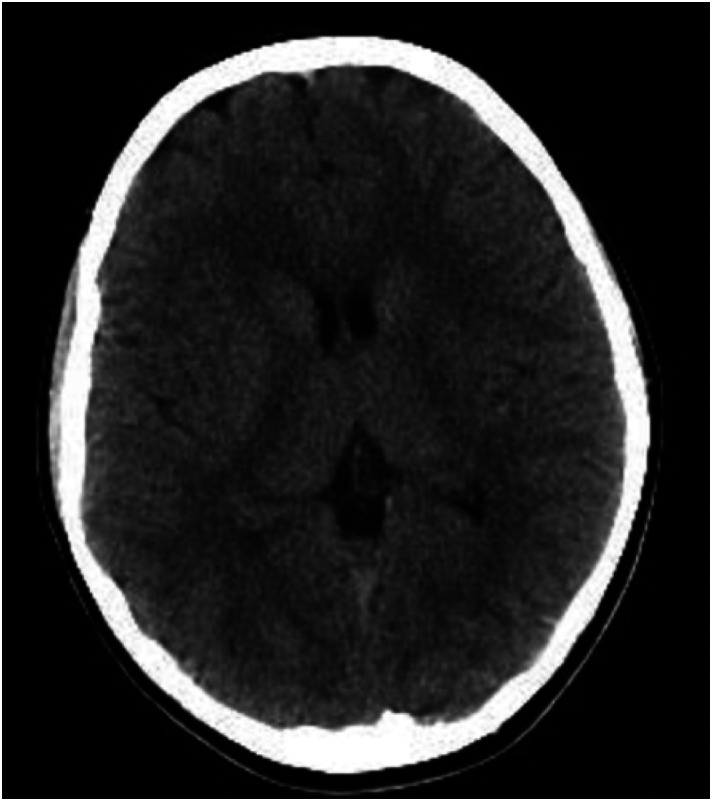
Normal non-contrast computed tomography of the brain.

**Image 2.**
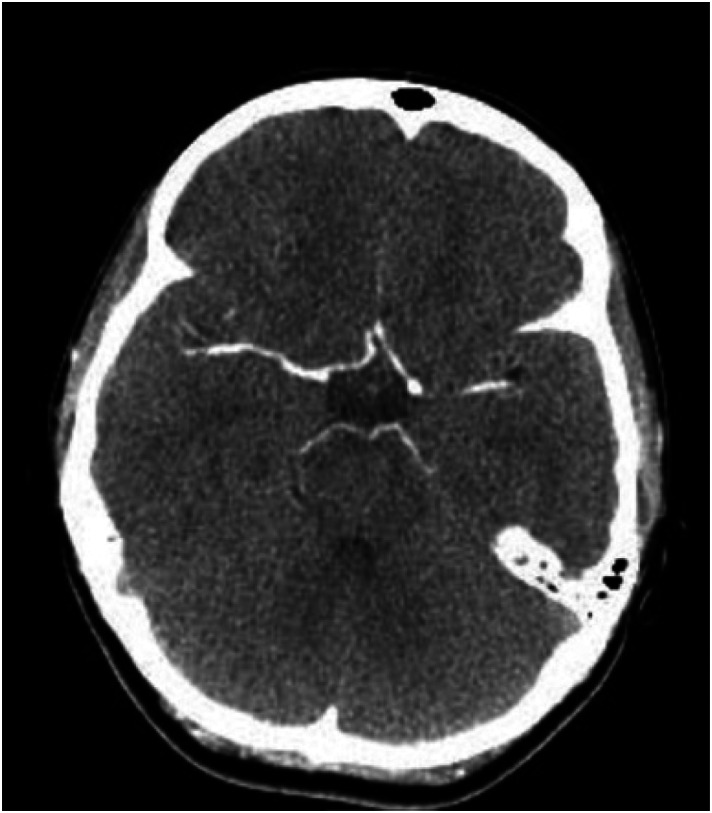
Normal computed tomography angiography at the level of the circle of Willis.

**Image 3.**
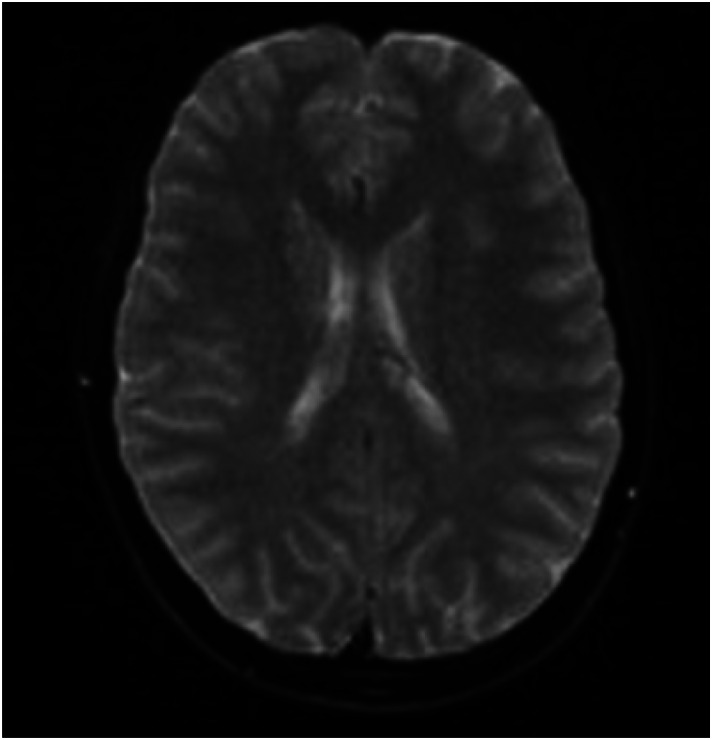
Normal magnetic resonance imaging of the brain.

Both a computed tomography (CT) (Image 1) and CT angiogram (CTA) (Image 2) of the brain were completed. No pertinent findings were yielded from the CTA; however, the non-contrast CT was read by neuroradiology as having a possible punctate focus of hemorrhage, which prompted further emergent imaging. This punctate focus was not appreciated by our team. A stat magnetic resonance imaging (MRI) of the brain was performed without significant findings (Image 3). Laboratory evaluation, including a complete blood count, comprehensive metabolic panel, pregnancy test, urine toxicology, and serum drug screen, were completed with no remarkable findings.

During the initial ED visit, the patient’s clinical status remained unchanged aside from an episode where she became somnolent, desaturating to 75% with good waveform and improving to 99% upon arousal. She appeared to be complaining of a headache and had one episode of vomiting; therefore, 1000 milligrams (mg) of acetaminophen and 4 mg of ondansetron were administered intravenously. Thrombolytics, such as tissue plasminogen activator or tenecteplase, were not administered throughout patient care, as neither have been studied in pediatric patients.^3^ Interfacility transport to a specialty pediatric hospital for pediatric neurology consultation and medical admission was arranged at that time.

During transport, all symptoms resolved aside from a headache and mild numbness of the right arm. Upon arrival at the pediatric ED, the patient was able to speak in complete sentences, and her NIHSS improved to 0. The patient was given a migraine cocktail of 25 mg ketorolac, 1000 milliliters normal saline, and 7 mg prochlorperazine intravenously. Following administration, the patient stated improvement in headache, thus concluding her symptoms, which in total lasted approximately 6.5 hours. Following a formal pediatric neurology consult, the working diagnosis was ACM. The patient was considered stable and was subsequently discharged that evening with an anticipated outpatient pediatric neurology appointment in one to two weeks.

## DISCUSSION

The diagnostic and treatment route chosen for this patient highlights the difficulty of clinical presentation to differentiate from other serious diagnoses, including cerebral infarction, intracranial hemorrhage, nonconvulsive status epilepticus, and encephalitis, among others.^1^ Ischemic pediatric strokes have an incidence rate of 1.2–13/100,000, making them exceedingly rare.^4^ However, with a 10% mortality rate and the critical consequence of neurological impairment in development, pediatric strokes are a serious medical emergency.^4^

Aside from astute history-taking, one of the few promising diagnostic tests to diagnose ACM is an electroencephalogram (EEG). In previous studies, EEG has shown slowing in 49 of 50 patients during the onset of symptoms through the retrospective study of patients with confusional migraines; therefore, we recommend the use of EEG in these patients, if capable.^1^ Beyond an EEG, no other diagnostic modality offers an indication for ACM until the benign resolution of symptoms, a characteristic of ACM known since the cases first recorded by Gascon and Barlow.^5^ Therefore, without any significant patient or family history beyond her mother’s transient global amnesia episode, or the capability of performing a spot EEG, a CT without contrast, CTA, and MRI were completed to rule out serious disease.

Prior to the onset of the patient’s ACM, the patient noted a fall backward in which she hit her head without loss of consciousness or vomiting. Although the pathophysiology has not been confirmed, literature review has shown that the onset of ACM is preceded by mild head trauma in 37% of patients.^1^ Some proposed mechanisms for ACM via mild head trauma include isolated cerebral edema and cortical spreading depression (CSD), inducing a temporary state of brainstem dysfunction.^6^ ^,^ ^7^ In these instances of mild, trauma-induced ACM, the typical time from traumatic injury to onset of ACM is seconds to approximately four hours, putting our patient’s fall well outside the typical timeframe of onset.^1^

While considering the CSD mechanism, in a retrospective study of 143 migraine patients 72.2% of those suffering migraines with aura reported deficits in higher brain function.^7^ With research demonstrating an association of migraine auras with CSD, it is plausible that the higher function deficits seen in cases of ACM are caused by CSD, thereby allowing ACM to be seen as a complex form of aura.^8^ However, with formal recognition and a standardized treatment for ACM lacking within the ICHD-3, the discussion now is whether ACM should have its own distinction.

Through retroactive studies it has been shown that of 2,509 diagnosed patients within a neuropediatric ward, 2.7% of the migraine diagnoses qualified as ACM.^9^ Without the specific classification criteria to diagnose these patients, diagnostic uncertainty can prevail, leading to misdiagnosis and underdiagnosis, especially amongst adults with a new diagnosis of migraine.^10^ Similarly, with the use of techniques such as CT, pediatric patients are put at a higher risk of malignancy following exposure to ionizing radiation.^11^ The use of CT highlights how diagnostic tools can potentially be hazardous, in addition to delaying diagnosis and treatment. Should a patient have a pertinent history for ACM, excessive diagnostics should be avoided, a viewpoint that has been held since ACM’s identification in the 1970s and well into the 21^st^ century.^12^ ^,^ ^13^

Should ACM earn classification within the ICHD-3, further formal research would likely occur to seek out a standardized treatment for episodes. To date, due to its rarity, knowledge of ACM is primarily limited to case reports, meaning standardized treatment has yet to be determined. Although, considering ACM as a migraine variant, case studies have shown success with traditional migraine treatment with sodium valproic acid.^14^ In this case, most symptoms self-resolved; however, the administration of a migraine cocktail including ketorolac, normal saline, and prochlorperazine did show improvement in the patient’s residual symptoms, supporting that traditional migraine treatments are effective for ACM.

It should be noted that although there is not enough research on ACM in particular, migraines are often associated with a variety of comorbidities, ranging from psychiatric disorders to epilepsy, as well as different means of cardiovascular compromise. For this reason, it is important to recognize as it may limit patient care options, and with future research could offer greater insight into the mechanism by which ACM arises.^15^

With the aforementioned in mind, it is important to consider the clinical approach for ACM. With a pediatric patient presenting with altered mental status, one must begin to rule out the broad spectrum of differential diagnoses. A complete set of vital signs, including rectal temperature (as appropriate) and blood glucose should be obtained. Thorough history-taking should be performed from the patient and/or any bystanders, to include relevant medical, surgical, psychiatric, and familial history. Care must be taken to also obtain potential ingestions, including those that are accidental, such as medications, drugs (edibles), environmental (carbon monoxide, chemical, cleaning products), etc. A thorough physical exam should elicit any signs of trauma, a complete neurologic exam, evaluation of the cardiac and respiratory systems, and a skin examination. The approach to laboratory testing should include the following, if relevant: complete blood cell count; complete metabolic panel; urinalysis; urine and serum toxicology studies; thyroid function tests; and cerebrospinal fluid testing. If concern exists for intracranial pathology, neuroimaging should not be delayed and a spot EEG can be performed, if available.

To pursue a route of treatment for a patient with suspected ACM, traditional migraine medications have shown promise. Treatment options may include the following: sodium valproic acid, prochlorperazine with or without diphenhydramine, metoclopramide, dihydroergotamine, triptans, ketorolac, steroids, or normal saline. Patients with ACM will classically see full resolution of symptoms without any lasting consequences after receiving treatment. Even with complete resolution, patients may be admitted for formal EEG monitoring or discharged with close outpatient neurology follow-up.

## CONCLUSION

Acute confusional migraines are a rare variant of migraine that primarily afflicts children. Lack of awareness of the disease, however, poses risks for delayed diagnosis, delayed treatment, and excessive diagnostic tests in pediatric patients. This case contributes to the growing collection of ACM episodes that continues to validate the argument for recognition of ACM by the ICHD-3. Clinically, ACM poses a challenge to diagnose; therefore, without a pertinent patient or family history, further diagnostics should be carried out to rule out serious disease. With cases of ACM, although the episodic symptoms are severe, physicians should find solace in knowing they are benign and self-resolving, and that traditional migraine treatments show promise in reducing or resolving symptoms.
